# The Antifungal Fibers of Polyamide 12 Containing Silver and Metal Oxides

**DOI:** 10.3390/ma16175837

**Published:** 2023-08-25

**Authors:** Paulina Latko-Durałek, Józef Rzempołuch, Monika Staniszewska, Karina Rosłoniec, Monika Bil, Rafał Kozera, Anna Boczkowska

**Affiliations:** 1 Faculty of Materials Science and Engineering, Warsaw University of Technology, Wołoska 141 Street, 02-507 Warsaw, Poland; jozef.rzempoluch.stud@pw.edu.pl (J.R.); rafal.kozera@pw.edu.pl (R.K.); anna.boczkowska@pw.edu.pl (A.B.); 2 Centre for Advanced Materials and Technologies CEZAMAT, Warsaw University of Technology, Poleczki 19 Street, 02-822 Warsaw, Poland; monika.staniszewska@pw.edu.pl (M.S.); karina.rosloniec@pw.edu.pl (K.R.); monika.bil@pw.edu.pl (M.B.)

**Keywords:** Polyamide 12, composite fibers, biocidal fillers, dispersion, melt spinning

## Abstract

The textile market is a vast industry that utilizes antimicrobial polymeric materials, including various types of fabrics, for medical and personal protection applications. Therefore, this study focused on examining four types of antimicrobial fillers, namely, metal oxides (zinc, titanium, copper) and nanosilver, as fillers in Polyamide 12 fibers. These fillers can be applied in the knitting or weaving processes to obtain woven polymeric fabrics for medical applications. The production of the fibers in this study involved a two-step approach: twin-screw extrusion and melt spinning. The resulting fibers were then characterized for their thermal properties (TGA, DSC), mechanical performance (tensile test, DMA), and antifungal activity. The findings of the study indicated that all of the fibers modified with fillers kill *Candida albicans*. However, the fibers containing a combination of metal oxides and silver showed significantly higher antifungal activity (reduction rate % R = 86) compared to the fibers with only a mixture of metal oxides (% R = 21). Furthermore, the inclusion of metal oxides and nanosilver in the Polyamide 12 matrix hindered the formation of the crystal phase and decreased slightly the thermal stability and mechanical properties, especially for the composites with nanosilver. It was attributed to their worse dispersion and the presence of agglomerates.

## 1. Introduction

In recent years, the world’s attention has been drawn to specific medical problems such as the COVID-19 pandemic outbreak or the severe threat of bacteria becoming resistant to antibiotics [1]. Moreover, some research confirms a fast spreading of the microorganism between non-protective objects, mostly in hospitals. It is because the equipment used is mainly made of typical thermoplastic polymers that are not resistant to the microorganisms. Therefore, bacteria, viruses, and fungi can multiply and accumulate quickly [2]. Due to these risks, a lot of research is focused on the modification of the polymers to achieve a material with antimicrobial properties [3,4,5].

To make the polymers resistant to microorganisms, they need to be modified with specific inorganic or organic fillers possessing antimicrobial properties. Among inorganic fillers, mainly metallic particles such as copper and silver are used; however, due to their high price, they are often applied in the form of oxides or nitrides. Silver is one of the fillers that is receiving the most attention, and can be used as an antibacterial, antifungal, or antiviral agent [6]. It can also improve the shelf life of food; therefore, it is used in the packaging industry. To improve the biocidal properties of silver, it is commonly used in the nanosize. However, it still needs to be thoroughly investigated whether and what adverse effects it may have on the human body. Some works describe that nanosilver affects the metabolic activity of cells, causes cell membrane damage, and eventually leads to cell death [7].

From the group of oxides, those based on copper, titanium, and zinc are also very popular biocidal additives. Titanium oxide particles (TiO_2_) like gold particles, exhibit photocatalytic activity [8] and their antimicrobial properties are induced by electromagnetic radiation from the visible light range, close to the UV range. This radiation induces the release of reactive oxygen species (ROS), which can interact with the cell membrane and DNA, damaging them and causing cell death. In addition, it is possible to enhance the antibacterial properties of TiO_2_ by combining it with silver nanoparticles. Zinc oxide (ZnO) particles are another inorganic filler with a biocidal effect. Studies have shown that ZnO is an effective antimicrobial agent against numerous microorganisms, and that its biocidal properties strongly depend on the particle size [9]. Its particles can interact with the cell membrane and generate ROS. Copper oxide (CuO) particles are other particles that show bactericidal capabilities. However, their properties were shown to be inferior to, for example, nanosilver, and a higher concentration of CuO particles than silver was needed to achieve the same effect [10].

The antimicrobial effect of the inorganic fillers can be explained based on two mechanisms. The first one relates to the reducing potential of metals by redox reactions, and the second relates to the interaction of metal ions with microorganisms. These ions can attach themselves in place of the metal particles initially present in the microbial cell, causing its destruction [11]. As with the inorganic particles, the antibacterial effect is achieved by interacting with the bacterial cell membrane and generating ROS. It is contrary to the organic fillers such as chitosan, polysiloxane, or triclosan in which the biocidal effect is achieved either through the release of antibiotics, proteins, or antimicrobial agents, or through a contact-acting cationic surface, such as ammonia compounds (second mechanism). Compared to inorganic fillers, the organic ones are less stable, especially at higher temperatures. It leads to difficulties in using them as additives in, for example, polymer composites, as the particles will not survive the manufacturing process, which is often carried out at high temperatures. For this reason, inorganic nanoparticles are used much more often than organic ones [12].

One of the sectors where antimicrobial polymeric materials are used is the textile market, including different types of fabrics dedicated to medical and personal protection applications. Most of these uses occur in the form of the nonwoven fabrics produced in three distinct stages, although advancements in technology have enabled some stages to overlap, and in certain instances, all three stages can occur simultaneously. The first stage involves web formation, which can vary depending on the raw material and technology used. This includes dry laid (carded or air laid), spun melt (spun bond, melt blown), wet laid, and others (electrospun, centrifugally spun, and self-assembled fibrous networks). The second stage is web bonding, which can be categorized into thermal (calendaring, air through), mechanical (needle punching, hydro-entanglement, stitch bonding), and chemical bonding. The final step involves the finishing treatment, which can include mechanical treatment, surface modification, or coating [13,14].

**Figure 1:**
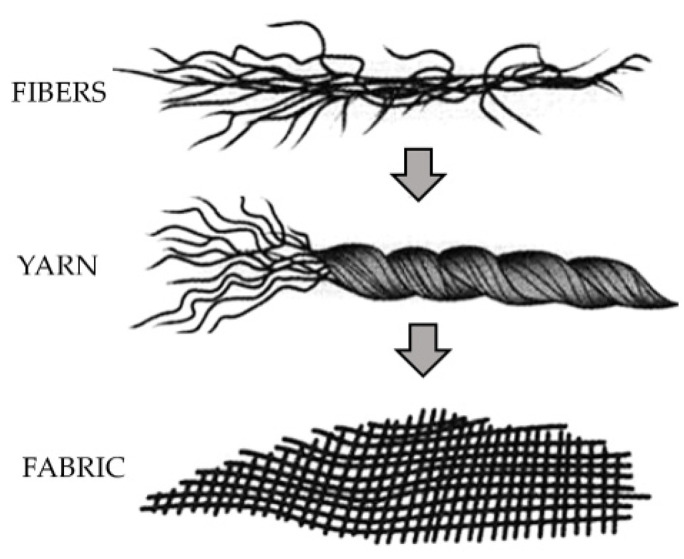
The difference between fiber, yarn, and fabric.

Contrary to the nonwoven fabrics, the woven ones are formed from yarns by their interlacing or intermeshing using the weaving and knitting processes, respectively. In order to create the yarns, the long single fibers (threads) need to be twisted and bonded together, as shown in Figure 1 [15]. So, the process of making functional fabrics starts with the fibers, which are modified towards the desired properties, such as the antimicrobial properties considered in this paper. It should be noted that the fabrics, due to their large specific surface area, combined with the tendency to absorb moisture, makes them a favorable environment for the proliferation of bacteria or fungi. Under optimal conditions (36–40 °C, pH 5–9), some species of bacteria can duplicate their numbers in 20–30 min [16]. Hence, there is justified attention towards developing woven functional fabrics with antimicrobial properties, which starts with fabricating the fiber.

Manufacturing antimicrobial composite fibers from the polymers can be achieved by directly mixing the fillers with the polymers or using solvent and forming the fibers by the melt-spinning process. The other method is a chemical bonding of antimicrobial agents to the surface of the neat fibers by chemical activation of the surface [17,18]. Teli and Kale studied the effect of different weight concentrations of ZnO (20–40 nm) on the antibacterial properties of PET matrix composite fibers [19]. Using a melt-spinning method, they produced fibers with 0.5 wt%, 1 wt%, and 1.5 wt% ZnO. It was observed that as the concentration increased, the antibacterial properties of the produced fibers increased, but at the same time, the mechanical properties decreased. Therefore, the optimal filler content was found to be 1 wt%. Shayestehfar et al. produced PA6 matrix fibers with different TiO_2_ additions having nanometer and micrometer scales [20]. The fibers were made using the melt-spinning method at concentrations of 0.03; 0.33; 0.5; 0.7 wt%. In this study, only the morphology of the fibers and their mechanical properties were examined. It was observed that there were numerous agglomerations of TiO_2_ particles; however, better dispersion occurred for the fibers containing nanometric TiO_2_. Perkas et al. produced PA6/nAg composite pellets by immersing pure PA6 pellets in a silver salt solution [21]. The result was a composite granulate with a silver content of 1 wt%, and the size of the silver particles varied between 50 and 100 nm. The nanocomposites produced in this way were processed into the form of fibers by melt spinning, and the resulting fibers showed good antibacterial properties.

In this paper, the composites fibers were produced by the melt-spinning process from Polyamide 12 and four types of the antimicrobial inorganic fillers based on the metal oxides (ZnO, CuO, TiO_2_) and on nanosilver. In our previous work [22], we examined the effect of the various concentrations of metal oxides (ACRAZ-172) on the PA12, and we found that the highest antimicrobial activity was at 3 wt%. Based on the results, that work aimed to compare the properties of PA12 fibers containing 3 wt% of metal oxides that differed in their composition. Separately, we compared two nanosilver types with different particle sizes, added at 1 wt%. It allowed identification of the most influential metal oxides and nanosilver, which increase the antifungal properties of Polyamide 12 fibers. It should be noted that the antimicrobial properties of polymer composites depend on the form of the specimens and will be different for the textile product and the bulk composites formed by injection molding. Here, we made an effort to analyze the antifungal properties of *Candida albicans* on thin fibers by quantitative analysis because fungi are the type of microorganism that is the most difficult to kill. Additionally, most of the papers focus on one type of the filler; the results presented in this work show a great innovation level connected with the practical application of functional Polyamide 12 fibers as a precursor of the woven fabrics.

## 2. Materials and Methods

### 2.1. Raw Materials

As the polymer matrix, Polyamide 12 (PA12) with the trade name Vestamid^®^ Z7321 (Evonik, Germany) and in the form of pellets was used. It has a density of 1.01 $\frac{g}{{\mathrm{cm}}^{3}}$ and relatively low water absorption. The recommended processing temperatures are in the range of 190–240 °C. As the antimicrobial fillers, four types of the fillers in the form of powders (delivered by Research and Development Center of Technology for Industry, Cracow, Poland) were used as listed in Table 1.

### 2.2. Fibers Manufacturing Process

**Figure 2:**
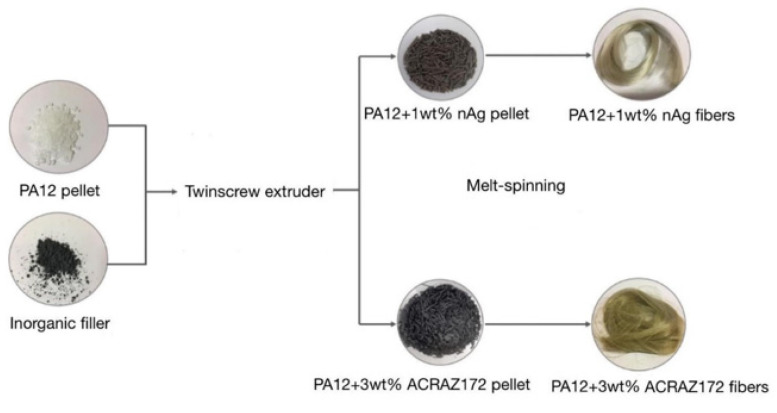
The schematic approach used to manufacture the composite fibers.

The composite fibers were fabricated by the two-step method schematically shown in Figure 2. Firstly, PA12 pellets were mixed with the powder of each of the fillers. ACRAZ-172 and ACRAZ-124 were added in 3 wt%, while nAg 100 nm and nAg 20–30 nm were added in 1 wt%. Components were mixed using a HAAKE MiniLab twin-screw laboratory extruder (Thermo Fisher Scientific, Waltham, MA, USA) at 190 °C and with screws velocity of 15 rpm. Low screws speed helped to achieved longer residence time helping to destroy the fillers’ agglomerates. In the second step, the formed pellets were extruded one more time using the same procedure to achieve homogenous dispersion of the fillers in the PA12 matrix. After mixing twice in this way, the composite pellets were obtained; the composite fibers were produced directly from these pellets using a melt-spinning process. For that, the twin screw extruder HAAKE MiniLab was connected with the transport belt and a homemade winding reel. By adjusting the speeds of each part (screws, belt, and reel) it was possible to produce fibers in a continuous process. Here, for each type of the filler, the melt-spinning parameters were kept the same: temperature of 190 °C, constant torque of 10 Ncm, and rotation of the winding reel at 1609 rpm. In the case of the fibers with the addition of ACRAZ-172 and ACRAZ-124, the process went without problems and the fibers were continuously produced. This was in contrast to the fibers containing both types of nAg, which had a high tendency to break off. As shown in Figure 2, the composite fibers containing only nAg were silvery, while the fibers with metal oxides had a more dirty yellow-like color.

### 2.3. Rheological Properties of Composite Pellets

The effect of the used fillers on the viscoelastic properties of PA12 was examined by the dynamic oscillatory test using the ARES 4400-0107 rheometer (Rheometric Scientific Inc., TA Instruments, New Castle, DE, USA) with a parallel-plate geometry mode. For that the round specimens having a diameter of 1.5 mm and a thickness of 2 mm were made by an injection molding machine. Firstly, the amplitude sweep test as a function of the variable strain γ (0.07–100%) at a constant frequency of 1 Hz was performed. From the linear elastic range of the storage modulus curves obtained during the amplitude sweep test, the strain of 10% was selected. With that strain, a frequency sweep test was performed at 190 °C with a frequency range from 0.1 to 100 Hz. The selected test temperature was the same as used during the extrusion process of the composites.

### 2.4. Microstructure Analysis

The dispersion of each type of the filler in the fibers was examined using a Scanning Electron Microscope (SEM) and a Scanning Transmission Electron Microscope (STEM). The SEM microscopy was also used to measure the fiber diameters. Samples for observation were prepared by sputtering using a POLARON SC7640 sputtering machine for 80 s at 1.5 kV. A Hitachi STEM S5500 microscope was used for high-resolution microstructure examination. Observations were conducted in a bright field at an accelerating voltage of 30 kV. To prepare the samples for testing, the samples were first encapsulated in epoxy resin, and then a thin film was obtained using a Leica EM UC6 ultramicrotome. The samples were cut at −80 °C, using a diamond blade. A film of approximately 75 nm thickness was obtained.

### 2.5. Thermal Properties

The thermal stability was analyzed by the Thermogravimetric (TGA) method using a TA Instruments TGA Q500. The samples prepared for the test had a mass of 9.5 ± 0.5 mg. The test was carried out under a nitrogen atmosphere, with a gas flow rate of 10 mL/min and 90 mL/min in the oven. Samples were heated from room temperature to 800 °C and 1000 °C at a heating rate of 10 °C/min. The resulting data were analyzed using TA Universal Analysis software. From the obtained curves, the degradation temperature at 5% (T_5%_) and 10% (T_10%_) weight loss, and the temperature of the maximum weight loss rate (T_d_), were determined.

Using the Differential Scanning Calorimetry (DSC) method, the melting point and crystallinity degree of the composite pellets and fibers were examined. The samples for the DSC test had a mass of 5.0 ± 0.1 mg. The test was carried out using TA Instruments’ DSC Q100 analyzer in three cycles: heating to 260 °C from room temperature, cooling to −90 °C, and heating to 260 °C. The temperature change rate was 10 °C/min and the helium flow rate was 25 mL/min. The data were analyzed using TA Universal Analysis software. The crystallinity content (*X_c_*) of the PA12 composites was calculated from the following Equation (1):

(1) $$X_{c}\left(\%\right)=\frac{\Delta H_{c}}{\Delta H{{}^{\circ}}_{m\;\;}\left(1-x\right)}\times 100\%$$

where:

Δ*H_c_* is the enthalpy of melting taken as the area under the melting peak from the second heating curve,

Δ*H^o^_m_* is the melting enthalpy of 100% crystalline PA12, which is 209 J/g [16],

*x* is the weight fraction of filler used.

### 2.6. Mechanical Properties

Dynamic mechanical analysis was performed by Dynamic Mechanical Analyzer DMA Q800 TA Instruments. Samples were tested using the Multi-Frequency–Strain mode at a constant frequency of 1 Hz. The testing temperature ranged from 25 °C to 180 °C and the heating rate was 3 °C/min. The data were analyzed using TA Universal Analysis software.

Mechanical testing was carried using the tensile machine IN-STRON 5943. The fibers (the gauge length of the sample was 60 mm) were subjected to tension at a rate of 20 mm/min. Six specimens of each type were tested to determine the average strength and elongation at break.

### 2.7. Antifungal Properties

Antifungal activity of PA12 composite fibers was performed using the AATCC TM100-2019 (The American Association of Textile Chemists and Colorists, AATCC TM100-2019, “Test Method for Antibacterial Finishes on Textile Materials: Assessment of”) [23]. Briefly, *C. albicans* ref. strain 90028 ATCC (LGC Standard, Warsaw, Poland) at 1 × 10^5^ CFU/mL of YEPD was incubated with the fibers (0.75 g) at 37 °C for 24 h [22]. Then, tenfold dilutions were plated on YEPD agar and the number of CFUs was counted for the tested and control (without any fillers) fibers. Reduction of *C. albicans* growth (% R) was calculated using the Equation: 100% × (A − B)/B, where A means CFUs recovered from the PA12 control inoculated with *C. albicans* and incubated over the 24 h contact period; B means CFUs recovered from the PA12 with metal oxide inoculated and incubated over the 24 h contact period. The GraphPad Prism 9 for Windows 64-bit v9.5.0 (730; 1992-2022 GraphPad Software Inc., La Jolla, CA, USA) was used for the data analysis. Differences between the groups were tested using the one sample t-test and Wilcoxon test. Differences were considered statistically significant at *p* < 0.05.

## 3. Results

### 3.1. Rheological Properties

**Figure 3:**
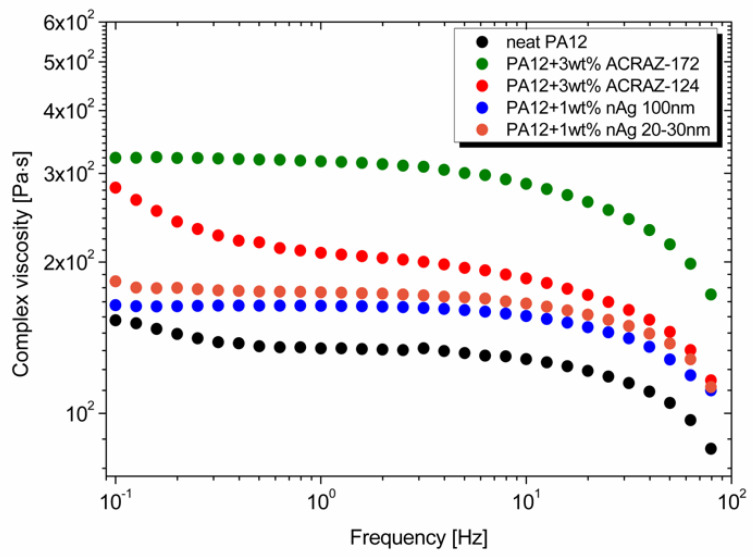
Complex viscosity of the PA12 composites in the function of frequency.

The oscillatory test analyzed the effect of the metal oxides and nanosilver addition on the viscoelastic properties of PA12. Figure 3 shows that all the materials behaved like Newtonian liquids at low frequency with constant viscosity. The viscosity dropped at frequencies higher than 10^1^ Hz, showing the shear-thinning behavior. Both types of antimicrobial fillers increased the viscosity of pure PA12 but at a low level. This was because the fillers have a spherical shape and were added at low concentrations. Interestingly, even though silver occurred in the nano size, it did not affect viscosity at such an excellent level as reported, for instance, for carbon nanotubes. This was due to a smaller aspect ratio and lower surface area. It was also confirmed by minor storage and loss modulus changes, as plotted in Figure 3. Furthermore, there was no effect of the specific silver size; both types with 20–30 nm and 100 nm showed similar behavior. When metal oxides were added, the viscosity of PA12 was higher, especially for ACRAZ-172. It could be related to its smaller size compared to ACRAZ-124. Moreover, metal oxides shift a characteristic shear-thinning behavior to a lower frequency than pure PA12 and composites with nAg.

**Figure 4:**
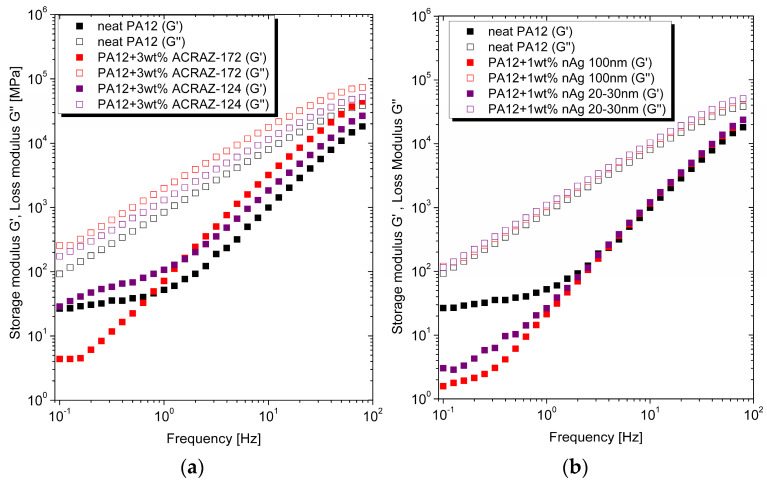
The dependence between storage (G′) and loss (G″) modulus of the studied composites containing metal oxides (**a**) and nanosilver (**b**).

For the unfilled polymer, the storage modulus was higher than the loss modulus, which points out that the material behaved more like viscous liquid (Figure 4a,b). That dependence remained unchanged after the addition of metal oxides (Figure 4a) and nAg (Figure 4b). It was because the used fillers possess a low aspect ratio so their interactions with the polymer chains was not sufficient to hinder the movement of the macromoelcules. From the practical point of view, the viscous response during the PA12 composites processing was more favorable than elastic which is generally expressed by a higher loss modulus [24,25].

### 3.2. Microstructure

The adjusted melt-spinning process parameters resulted in the formation of fibers containing various biocidal fillers. According to the SEM analysis and results collected in Table 2, the neat PA12 fibers had a diameter of around 80 mm with the smallest standard deviation. In the presence of the fillers, as the process conditions changes, the fibers generally had higher diameters. However, the addition of metal oxides (ACRAZ-172 and ACRAZ-124) did not affect the formation of the fibers much; therefore, the measured diameters were around 80 mm. In the case of nanosilver, the fibers had higher diameters of about 100 mm.

**Figure 5:**
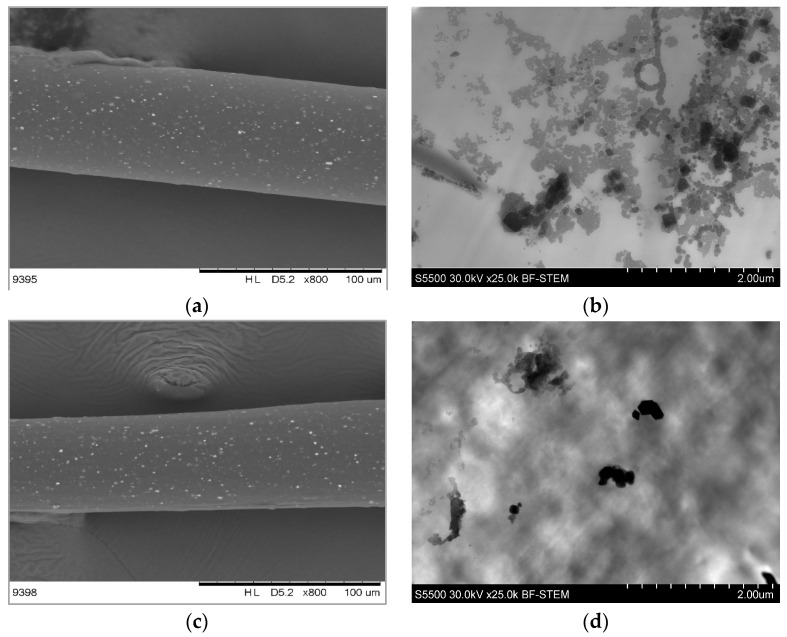

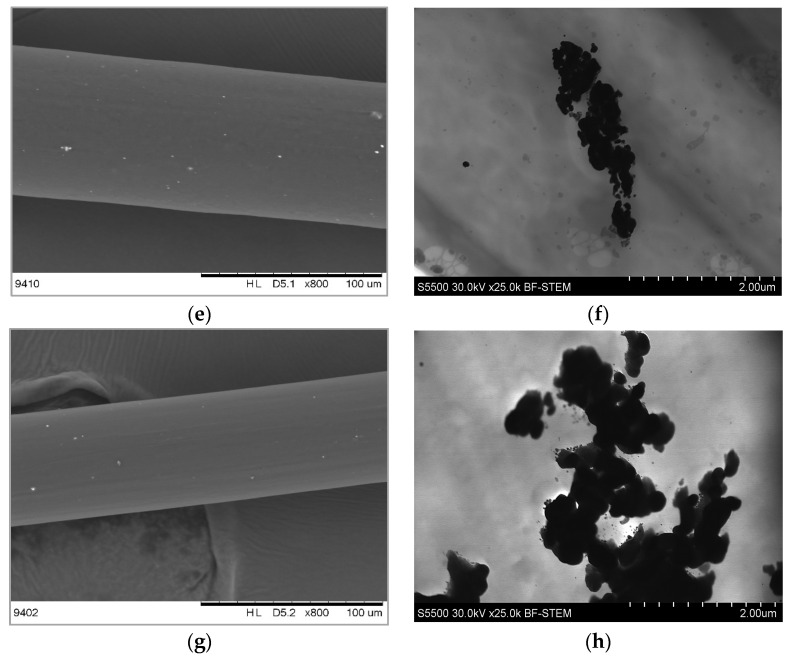
The dispersion of the biocidal fillers in PA12 fibers containing: 3 wt% ACRAZ-172 (**a**,**b**); 3 wt% ACRAZ-124 (**c**,**d**); 1 wt% nAg 100 nm (**e**,**f**) and 1 wt% nAg 20–30 nm (**g**,**h**).

Looking into the images taken by the SEM, the dispersion of each filler in the fiber’s length was analyzed (Figure 5, left column). It can be easily observed that the added fillers were visible as white dots. They were homogeneously dispersed in the whole fiber when the metal oxides were added as the filler, while the addition of silver resulted in poor dispersion with only a few places where the filler could be found. Analyzing the images taken with a high-resolution microscope (Figure 5, right column), it was found that nanosilver in the fibers occurred in the form of the agglomerates (black clusters). Especially for the silver particles with a size of 20–30 nm, the agglomerates were the biggest (Figure 5h). This is consistent with the theory that particles of a smaller size show a greater tendency to agglomerate [26].

In the case of ACRAZ-172 and ACRAZ-124, the oxides were much better dispersed; however, the fibers contained the particles of ACRAZ-172 (Figure 5b), and there were some agglomerates, but they were smaller than those found in the fibers with nanosilver (Figure 5f,h). For the fibers with ACRAZ-124, the metal oxides were well-dispersed, but single agglomerates were also found. They were formed by the silver which was included in the ACRAZ-124 filler, rather than by the metal oxides. Achieving more homogenous dispersion for the metal oxide fillers was linked to their concentration that was higher (3 wt%) than for nanosilver (1 wt%) and because the particles were bigger. That caused higher viscosity and more effective shear force during extrusion, which positively affected the dispersion of the particles in the polymer matrix [27].

### 3.3. Thermal Properties

**Figure 6:**
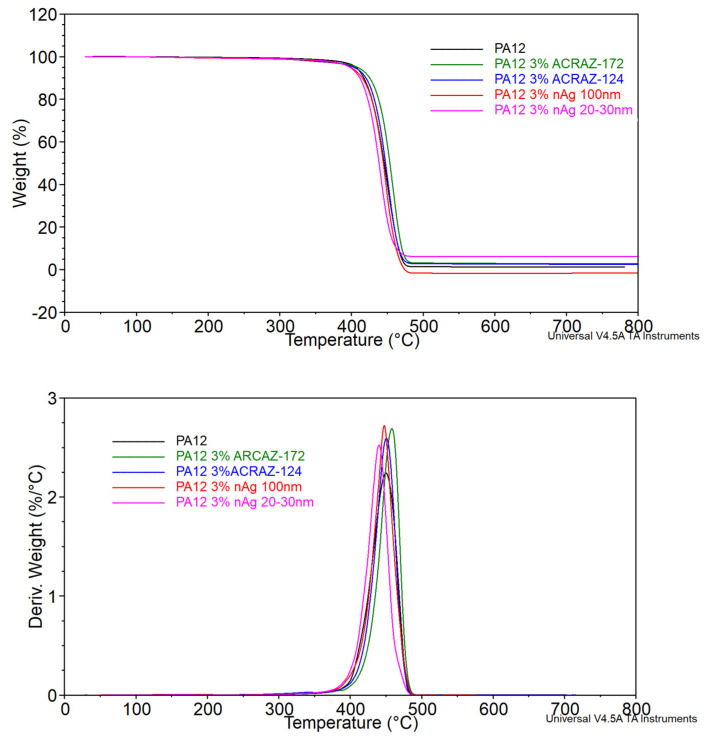
TG and DTG curves of the studied PA12 composites.

**Figure 7:**
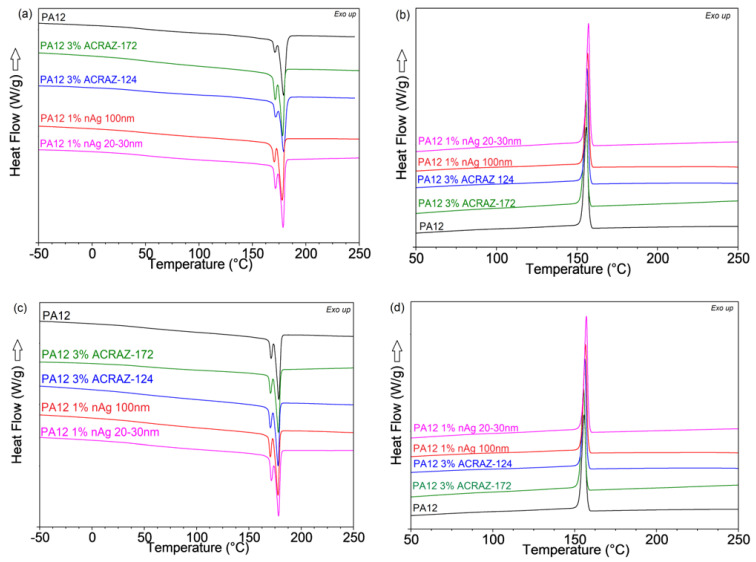
The second heating and cooling curves obtained from the DSC analysis for composite pellets (**a**,**b**) and fibers (**c**,**d**) containing biocidal fillers.

The results of the TGA and DSC tests are summarized in Figure 6 and Figure 7 and Table 3. The TGA analysis revealed that the decomposition temperature (T_5%_) for the composites with metal oxides was a few degrees, which could be negligible. Although, in the case of the composites containing nAg, the values of T_5%_ were lower at about 7 and 8 °C compared to pure PA12. The highest degradation rate (T_d_) temperature and the percentage of the material decomposing at that temperature were higher than for the pure PA12. It means that the overall thermal stability of PA12 in the presence of antifungal fillers was decreased. It could be related to their low concentration or poor dispersion and, by this, the lack of adsorption of polymer chains to the surface of the filler particles responsible for increasing the thermal stability in the polymer composites. The other possibility is the concentration of metal ions [28].

Figure 7 presents the melting and crystallization behavior of the tested composite pellets and the fibers collected during the second heating and cooling cycles in the DSC test. First, it is visible that a single melting point occurred for the neat PA12 pellet splits to the double peak after the addition of the metal oxides and nAg (Figure 7a). That should refer to the formation of a phase that melts at a lower temperature of around 168 °C. It is not a result of the fillers addition but the processing by extrusion at 190 °C because such a double melting peak was also detected for the fibers of the unfilled PA12 shown in Figure 7c. The formation of the α-phase in PA12 and its transition into the γ-phase, which melts at a higher temperature of 178 °C, has been described in the literature [29]. Since the researchers reported that at a temperature above 80 °C, only the γ-phase occurs, it may be concluded that in the presence of biocidal fillers, the transition of the α-phase into the γ-phase is constricted [30]. Because of that, there is a negligible change in the crystallinity content for the pure processed PA12 and its composites containing metal oxides and nAg. A lack of nucleation phenomena is also confirmed by the unchanged crystallization curves for the pellets and fibers present in Figure 7b,d, respectively. In the case of the fibers, the applied stress during the melt-spinning process was not enough to induce the formation of the ordered macromolecules chains [31]. Furthermore, it could also be attributed to the hindered movement of macromolecules during the melt-spinning process resulting in a non-ordered structure [32].

### 3.4. Mechanical Properties

**Figure 8:**
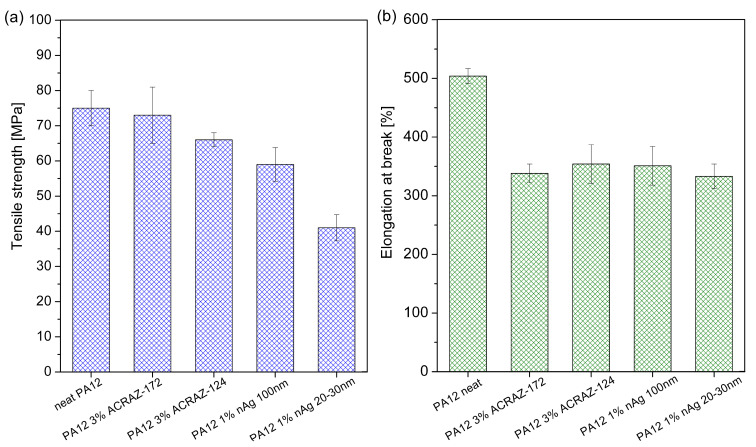
Mechanical properties of PA12 fibers filled with biocidal fillers: (**a**) tensile strength and (**b**) elongation at break.

The tensile test results revealed that incorporating the biocidal fillers decreased the tensile strength (Figure 8a) and elongation at break (Figure 8b) of the PA12 fibers. For the PA12 pure fibers, the tensile strength was 75 MPa, and after the incorporation of ACRAZ-172 and ACRAZ-124, it slightly decreased to 74 MPa and 67 MPa, respectively. In the case of nAg, the fibers had much lower strength that dropped to 59 MPa for nAg with 100 nm and 41 MPa for nAg with a size of 20–30 nm. Such reduction of tensile strength was due to the non-uniform distribution of the incorporated particles and the presence of agglomerates within the polymer matrix, which the SEM and STEM analysis confirmed (see Figure 5f,h) [26]. In Figure 7b, the elongation results at the break of the fibers were collected. Unfortunately, all the fillers decreased the elongation by about 200% compared to pure PA12. Therefore, it can be concluded that the biocidal fillers did not provide a reinforcing effect and caused the PA12 fibers to become more brittle.

**Figure 9:**
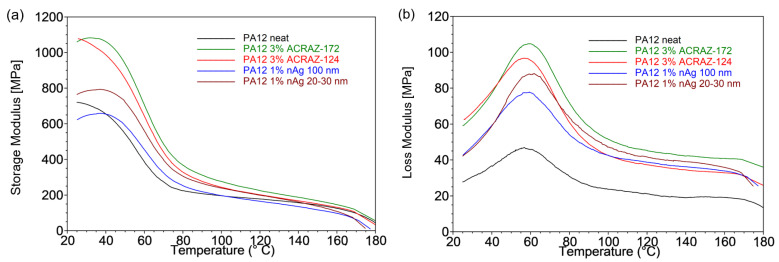
The storage (**a**) and loss modulus (**b**) in the function of temperature for the analyzed composites fibers.

The variation of the viscoelastic properties of the fibers as the function of temperature at a frequency of 1 Hz is given in Figure 9. It is visible that both the storage (E′) and loss moduli (E″) were affected by the type, content, and size of the used fillers. The storage modulus of the composites in the glassy region increased significantly after the incorporation of ACRAZ-172 and ACRAZ-124 to the values of 1082 MPa and 1024 MPa, respectively. nAg with 20–30 nm particles also resulted in a higher E′ than for the neat PA12, but the achieved value (E′ = 798 MPa) was lower than for the fillers based on the metal oxides. The lowest storage modulus was determined for the fibers filled with nAg 100 nm (E′ = 679 MPa). The increasing value of E’ indicated the growing stiffness of PA12 loaded with ACRAZ-172 and ACRAZ-124 could be related to their higher concentration, thus promoting more effective stress transfer, which resulted in the stiffness increasing and hence a higher storage modulus [33]. However, as the storage moduli curve transitioned from a glassy state to a rubbery plateau state above 65 °C, these differences between E′ became negligible [34]. Similarly, the loss modulus (E″) that correlated with the viscous properties of a polymer matrix also increased after the addition of the biocidal fillers. The neat PA12 fibers exhibited the lowest value of the loss modulus peak at 49 MPa, which was twice as high for the fibers containing nAg 100 nm (E″ = 80 MPa) and ACRAZ-172 (E″ = 110 MPa). An increase in the loss modulus was reported by other authors and was correlated with lower mobility of the macromolecule chains in the composites [35]. It is caused by good fiber–matrix bond strength, which occurred for ACRAZ-172 and ACRAZ-124.

### 3.5. Antifungal Properties

**Figure 10:**
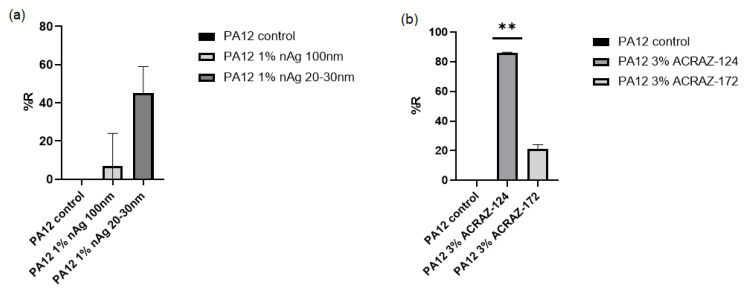
The antifungal activities of PA12 fibers containing nanosilver (**a**) and metal oxides (**b**) in terms of reduction (% R) rate of *C. albicans* CFUs vs. PA12 fibers control (without any fillers). Differences with *p* < 0.05 were considered statistically significant. Two asterisk (**) indicates *p* = 0.0026.

The main role of the added fillers was to make the PA12 fibers able to kill the microorganisms. Hence, the produced fibers were tested against example fungi—as they are the most difficult microorganisms to be destroyed. The results collected in Figure 10 show the standard quantitative procedure (AATCC TM100-2019, [22]) of the antifungal activity of the PA12 fibers containing metal oxides or silver nanoparticles vs. the PA12 control (without any fillers). Comparing the fibers with nAg (Figure 10a), it is seen that higher biological activity was obtained for the particles with a smaller size (20–30 nm) for which the reduction rate (% R) of *C. albicans* CFU was 45 (*p* = 0.137), while for the fibers with nAg 100 nm, % R = 7 (*p* = 0.664). This is related to the greater surface area and closer interactions with the microorganisms. For fibers with metal oxides, those with ACRAZ-124 possessed significantly higher activity than the fibers with ACRAZ-172. The fibers of PA12 + 3 wt% ACRAZ-124 showed the most effective reduction rate (% R) of *C. albicans* CFUs equals %R = 86; *p* = 0.0026. For the metal oxides which do not contain silver in their composition (ACRAZ-172), the anti-*C. albicans* activity was lower (% R = 21; *p* = 0.06). The better performance of ACRAZ-124 could be associated with its homogenous dispersion but also due to the presence of silver.

## 4. Conclusions

This paper presents the composite fibers of PA12 and four types of fillers based on metal oxides and nanosilver that possess biocidal properties. In the presence of the fillers, the applied melt-spinning process was the most difficult in the case of the nanosilver particles; therefore, the fibers often broke. Therefore, their diameter was around 100 mm, while for the fibers containing only metal oxides, it was approximately 80 mm. The worse mechanical properties of the fibers with nanosilver were expressed by the lowest tensile strength of 40 MPa which was twice as low than for pure PA12. Compared to the fibers with nanosilver, those containing metal oxides were characterized by a similar strength as the neat PA12 fibers and improved stiffness. The viscoelastic properties analyzed by the DMA were improved only for the fibers with metal oxides. They also showed slightly higher thermal stability, but there was no difference in their crystallization behavior. All the types of fillers did not promote the formation of a new crystal phase because the crystallinity content was almost the same for pure PA12 and its composite pellets and fibers. Analyzing the microstructure of the fibers using a high-resolution microscope, it was found that the metal oxides were homogeneously dispersed in the fibers. The antifungal activity against *C. Albicans* showed the highest effectiveness of the fibers containing the mixture of metal oxides and silver (% R = 80) than only the pure metal oxides. For the fibers modified with nanosilver, a higher %R was achieved for the smaller-sized particles. Due to the good mechanical and biocidal performance, the composite fibers of PA12 are a promising precursor for the manufacturing of woven fabrics.

## Figures and Tables

**Table 1:** Types of the antimicrobial fillers used.

Name	Composition	Particles Size
ACRAZ-172	mixture of CuO, ZnO, TiO_2_	300 nm—20% 500 nm—30% 1000 nm—50%
ACRAZ-124	mixture of CuO, ZnO, TiO_2_, Ag	200–2000 nm
nAg 100 nm	nanosilver	100 nm
nAg 20–30 nm	nanosilver	20–30 nm

**Table 2:** The average diameter of the fibers produced.

Fiber	Average Diameter [μm]
PA12 neat	82.3 ± 7.90
PA12 + 3 wt% ACRAZ-172	79.9 ± 25.1
PA12 + 3 wt% ACRAZ-124	73.2 ± 21.9
PA12 + 1 wt% nAg 100 nm	109 ± 10.5
PA12 + 1 wt% nAg 20–30 nm	102 ± 16.7

**Table 3:** The results of TGA and DSC analysis.

TGA			DSC						
Material	T_5%_ (°C)	DTG 1 (°C; %/min)	Pellets				Fibers		
			T_g_ (°C)	T_m_ (°C)	X_c_ (%)	T_c_ (°C)	T_m_ (°C)	X_c_ (%)	T_c_ (°C)
PA12	406	450; 2.24	49.5	178	25.4	149	177	26.9	155
PA12 + 3 wt% ACRAZ-172	408	459; 2.69	50.8	178	27.8	156	178	24.5	155
PA12 + 3 wt% ACRAZ-124	404	451; 2.57	50.6	179	26.8	157	178	27.3	156
PA12 + 1 wt% nAg 100 nm	399	449; 2.69	50.5	178	25.1	156	178	24.3	157
PA12 + 1 wt% nAg 20–30 nm	398	440; 2.52	49.9	179	25.2	156	178	26.5	157

## Data Availability

https://www.mdpi.com/2073-4360/14/15/3025, accessed on 11 July 2023.
